# 1,25-dihydroxyvitamin D as Predictor of Renal Worsening Function in Chronic Kidney Disease. Results From the PASCaL-1,25D Study

**DOI:** 10.3389/fmed.2022.840801

**Published:** 2022-03-02

**Authors:** Andrea Galassi, Eliana Maria Fasulo, Paola Ciceri, Roberta Casazza, Fabrizio Bonelli, Claudia Zierold, Mariella Calleri, Frank A. Blocki, Maria Assunta Palmieri, Claudio Mastronardo, Mario G. Cozzolino

**Affiliations:** ^1^Renal Division, Azienda Socio Sanitaria Territoriale (ASST) Santi Paolo e Carlo, Department of Health Science, University of Milan, Milan, Italy; ^2^DiaSorin SpA, Saluggia, Italy; ^3^Consultant for DiaSorin, Lugano, Switzerland; ^4^DiaSorin Inc, Stillwater, MN, United States; ^5^Reply Data, Milan, Italy

**Keywords:** CKD, 1, 25-dihydroxyvitamin D, PTH, PTH (1–84), vitamin D, tubular biomarkers, CKD-MBD

## Abstract

**Background:**

Heterogeneous progression of chronic kidney disease (CKD) toward dialysis advocates improving in renal care management. Diagnosis and staging of CKD relies on estimated glomerular filtration rate (eGFR) and albuminuria. Tubular biomarkers emerged as new predictors of worsening renal function (WRF), due to partial inaccuracy of eGFR and existing WRF in non-proteinuric patients. Active vitamin D is synthesized in renal tubules and participates to mineral adaptation in CKD. Circulating 1,25-dihydroxyvitamin D [1,25(OH)_2_D] was poorly investigated as a biomarker of endocrine tubular function and predictor of WRF.

**Objective:**

Investigate capability of 1,25(OH)_2_D to predict parathormone (PTH) increase and WRF in CKD stage 3–4.

**Methods:**

PASCaL-1,25D was an observational, prospective, monocentric study. Primary outcomes were absolute and 20% increase in PTH, and WRF defined as 20% reduction in eGFR or dialysis initiation at 6 months.

**Results:**

Seventy-one patients completed follow up. Absolute increase in PTH (1–84) was independently predicted by lower 1,25(OH)_2_D levels (*p* = 0.0134). No association was detected between 1,25(OH)_2_D and iPTH increase. Higher 1,25(OH)_2_D was associated with reduced risk of WRF at univariate analysis [OR 0.89 (95% CI 0.86–0.93), *p* = 0.006]. The 1,25(OH)_2_D/PTH (1–84) ratio was associated with non-significant 84% risk reduction for WRF [OR 0.16 (95% CI 0.06–0.41), *p* = 0.05]. Low 1,25(OH)_2_D reached 100% sensitivity in predicting WRF in CKD stage 3 (AUC 9.909, *p* < 0.0001) and non-elderly patients (AUC 0.883, *p* < 0.0001). Machine learning models retained 1,25(OH)_2_D/PTH (1–84) as relevant predictor of WRF together with eGFR and albuminuria. Age influenced interaction between renal and mineral biomarkers.

**Conclusion:**

1,25(OH)_2_D deserves attention as biomarker of tubular health, and sensible predictor of WRF on the short run among non-elderly patients affected by stage 3 CKD. The 1,25(OH)_2_D/PTH (1–84) ratio may represent a composite biomarker of tubular reserve/endocrine response to the transition from adaptive to maladaptive equilibrium in CKD-MBD.

## Introduction

Chronic kidney disease (CKD) represents a major public health problem, associated with poor quality of life, reduced survival and considerable economic impact (1–3). Recognizing individuals at risk for progression toward end stage renal disease (ESRD) remains challenging, although being essential to prevent patients from advancing to dialysis and renal transplant (4, 5).

Stratification of the risk for CKD progression currently relies on estimated glomerular filtration rate (eGFR) and albuminuria (6). However, their accuracy is limited by intrinsic unreliability of serum creatinine (sCr) and by the existence of non-albuminuric patients, who proceed toward ESRD (7, 8). Furthermore, eGFR and albuminuria do not reflect tubular injury (9), which on the contrary was associated with the risk of worsening renal function (WRF) in biopsy proven CKD (10, 11).

Although biomarkers of tubular damage are emerging as new predictors of WRF (12–16), a gold standard for non-invasive assessment of tubular health has not been validated. Nonetheless, renal tubules respond to alterations in mineral homeostasis secondary to lowered GFR. Hydroxylation of 25 hydroxyvitamin D [25(OH)D] into the active form 1,25-dihydroxyvitamin D [1,25(OH)_2_D] by the renal 1-α-hydroxylase (CYP27B2) represents a major endocrine function of the proximal renal tubule. Expression of CYP27B2 is up and downregulated by parathormone (PTH) and fibroblast growth factor-23 (FGF-23) respectively in the context of chronic kidney disease and mineral bone disorder (CKD-MBD) (17). Hence, it could be argued that CYP27B2 activity might be taken as biomarker of tubular endocrine reserve and of homeostatic response to mineral derangement.

Expression of 1-α-hydroxylase (CYP27B2) declines in CKD, secondary to the loss of nephron mass and to the direct inhibition elicited by increasing levels of FGF-23 (18–20). Consequent reduction in 1,25(OH)_2_D triggers the compensatory PTH increase, leading to secondary hyperparathyroidism (SHPT) (21, 22). In the impossibility to directly assess CYB27B2, circulating levels of 1–25(OH)_2_D might be considered a proxy of tubular synthesis, and 1,25(OH)_2_D/PTH ratio as a composite marker of tubular endocrine reserve and setpoint of transition from adaptive to maladaptive response in CKD-MBD.

It has been suggested that a composite risk factor, derived from the aggregation of both tubular synthesis biomarkers and hormones involved in mineral metabolism, may predict WRF. *Post-hoc* analysis from the SPRINT trial reported on how intact PTH (iPTH) and FGF-23, included with uromodulin (UMOD) into a single tubule reserve/mineral metabolism factor, resulted as unique independent predictor of eGFR decline (13). Thus, 1,25(OH)_2_D and 1,25(OH)_2_D/PTH ratio might deserve attention as negative risk factors of WRF.

However, reliable assessment of 1,25(OH)_2_D and of biologically active PTH remains an unmet need in clinical practice (23–25). DiaSorin LIAISON® XL analyzer is a validated and fully-automated method, which provides both the-third generation assay for the only biologically active PTH (1–84) whole peptide (26) and the accurate assessment of 1,25(OH)_2_D (27, 28). The 1,25(OH)_2_D/PTH (1–84) ratio, measured by aforementioned assay, independently predicted WRF in heart failure (29) and CKD stage G3b to G5 patients (30).

Results of PASCaL-1,25D study (PredictAbility of Secondary hyperparathyroidism and Ckd progression by circulating Levels of 1–25(OH)_2_ vitamin D) are herein presented. The study was designed to investigate the capability of low 1,25(OH)_2_D levels in predicting WRF and PTH increase, among adult patients affected by CKD stage G3 to G4 during 6-months follow up.

## Methods

### Study Design

PASCaL-1,25D study was an observational, prospective, monocentric study, conducted from September 2017 to November 2018 at the Renal Unit of San Paolo Hospital in Milan (Italy).

All adult patients, attending follow up at Renal Clinic due to CKD stage G3 to G4, were enrolled from September 2017 to May 2018. Subjects receiving nutritional vitamin D or vitamin D receptor activators (VDRA) were included. Exclusion criteria were age ≥85 or age <8 years, primary hyperparathyroidism, previous parathyroidectomy, pregnancy, lactation, ongoing bisphosphonates, denosumab, teriparatide, strontium ranelate, and inability or unwilling to subscribe written informed consent.

Primary outcomes consisted in: analyzing capability of basal 1,25(OH)_2_D to predict absolute and 20% increase in PTH (1–84) and iPTH levels at 6-months, and investigating basal 1–25(OH)_2_D as predictor of WRF, defined as 20% reduction in eGFR or dialysis initiation at 6-months follow up.

Secondary objectives consisted in: correlating 1,25(OH)_2_D with eGFR and measured GFR (mGFR) at baseline, and correlating baseline 1,25(OH)_2_D to relevant analytes in the context of CKD-MBD, including iPTH, PTH (1–84), 25(OH)D, serum calcium (sCa), serum phosphorus (sP), intact FGF-23 (iFGF-23), bone specific alkaline phosphatase (BSAP) and sclerostin.

Exploratory analysis was included, for testing 1,25(OH)_2_D/PTH (1–84) and 1,25(OH)_2_D/iPTH ratios as predictors of 6-month WRF.

Age, gender, mGFR, history of cardiovascular disease (CVD), diabetes, arterial hypertension, cancer and the cause of CKD were collected at baseline. Following clinical and biochemical data were recorded at study entry, 3 and 6 months clinical visits: height, weight, blood pressure, heart rate, ongoing therapies for hypertension and diabetes, prescription of nutritional vitamin D and VDRA, sCr, 25(OH)D, 1,25(OH)_2)_D, PTH (1–84), iPTH, total sCa, sP, iFGF-23, sclerostin, alkaline phosphatase (ALP), bone specific alkaline phosphatase (BSAP), glycated hemoglobin (Hba1c) in diabetic patients, 24 h urinary analysis for creatinine, albumin, sodium, P and Ca. Medical therapies, including vitamin D, were modifiable during study period, based on nephrologists' clinical judgement.

Seventy patients were estimated as the sample size required, for detecting 20 PTH increase and 20% WRF at 6 months with 95 significance and 80% study power, based on 15–25% prevalence of CKD stage G3 to G4 observed at Renal Unit of San Paolo Hospital during a 2-months pre-study investigation (data not shown). Patients were censored whenever lost to follow up or due to voluntary withdrawal from the study.

Estimated GFR was assessed by CKD-EPI formula (31) at baseline, 3 and 6 months. Measured GFR was also assessed at study entry by 99 mTc-diethylenetriamine-pentaacetic acid renal scintigraphy, performed at the Nuclear Medicine Unit of San Paolo Hospital (32).

All the blood samples were collected at fasting between 7:00 and 10:00 a.m. Routine biochemical measurements were performed at the corresponding local accredited laboratories by standardized assays. Assessment of bone mineral biomarkers was performed at DiaSorin Inc. (Stillwater MN, USA) on a LIAISON® XL analyzer using the fully-automated LIAISON® assays for serum 1,25(OH)_2_D (#310980), total 25(OH)D (#310600), plasmatic PTH (1–84) (#310630), plasmatic iFGF-23 (#318700), serum sclerostin (#310930), and BAP OSTASE® (#310970). Samples for immunoassays assessment were prepared for testing and frozen (−20°C), until testing, conducted at DiaSorin laboratories (Stillwater, MN). All other blood and urinary analysis were performed at the San Paolo Hospital Laboratory by standardized routine assays. Second-generation assay, adopted for iPTH assessment, was t by VITROS 5600 integrated system.

PASCAL-1,25D study was approved by San Paolo Hospital ethical committee.

### Statistical Analysis

Categorical and continuous variables were reported as mean ± SD or median (IQR) and frequencies (%) as appropriate. Patients' characteristics at baseline were stratified according to: basal eGFR (higher-equal or lower than 30 ml/min/1.73 m^2^), WRF and 20% increase in PTH (1–84). Differences between strata were tested for significance by ANOVA or Mann-Whitney U Test and Chi-squared or Fisher-exact test for continuous and categorical variables, respectively, as appropriate for normality of distribution.

Predictability of absolute and 20% increase in PTH (1–84) and iPTH was tested by separated multivariate regression models. Backward elimination procedure was adopted for best model selection. The following parameters were included as predictors at first step, based on clinical judgment in addition to the variables associated with PTH increase at univariate analysis: age, gender, 1,25(OH)_2_D, 25(OH)D, basal eGFR, any vitamin D therapy along the study period, urine albumin creatinine ratio (uACR) as continuous variable, iFGF23, sclerostin, sCa, sP, BSAP, diabetes and body mass index (BMI). Secondary models were performed, adopting 1,25(OH)_2_D as categorical variable, stratified according to threshold derived by Receiver Operating Characteristic analysis (ROC).

Univariate regression models were built to assess the association between 20% WRF and biochemical predictors. ROC analysis was performed to predict 20% WRF by 1,25(OH)_2_D, and eGFR in the whole cohort. DeLong and binomial exact methodology were applied to ROC analysis, which was further stratified according to basal eGFR and elderly.

Repeated measure analysis of variance was applied, for comparing renal and mineral parameters between baseline and 6-month follow up, stratified according to basal eGFR and to the presence of any ongoing vitamin D therapy.

Linear correlation between GFR, 1,25(OH)_2_D and PTH (1–84) at baseline was assessed by Pearson coefficient analysis. Spearman correlation matrix was performed between parameters of renal function and mineral metabolism at baseline, stratified according to age.

Classic statistical analysis was conducted by the programs R and MedCalc v20.

Non-prespecified *post-hoc* analysis was performed by machine learning (ML) techniques, for compensating unbalance derived by the low rate of WRF events observed. XGBoost methodology was applied to train a regression model in a supervised setting, for ascertaining which biomarkers may elicit biggest impact in predicting eGFR at 3-months follow up (33–36). In the training phase, 80% of the dataset was used to train the algorithm. The performance of resulting model was further tested on the remaining 20% of the dataset. Since XGBoost and other ML algorithms rely on a set of hyper parameters to optimally solve the machine learning problem, an extensive hyper parameter tuning phase was performed (37). A grid search, guided by squared error metric over 2-fold cross validation, was used to selected best values for each hyper parameter. GridSearchCV API from the scikit-learn package was adopted in conjunction with the XGBRegressor API from the XGboost library (38). The resulting values were 0.1 for the learning rate, 4 for the trees max depth, 0.7 for the observations subset sampling rate of each tree and 50 for the number of XGboost estimators. The final XGBoost. The model was trained by with these hyper parameters values. Model was evaluated on the test set by the mean absolute percentage error (MAPE) (39), which represents the percentual absolute error of model prediction. The scaling compared to the actual value allowed to properly weigh errors for low values of eGFR (39). Variables were finally sorted, according to their single weight in eGFR prediction after ex-post analysis.

## Results

A total of 74 patients were enrolled, of whom three were subsequently lost to follow up. Analysis was performed on 71 patients.

In the overall population median age was 75 years (69–80 IQR), with 73% patients being older than 70 years (Table 1). Baseline eGFR and urinary albumin to creatinine ratio (uACR) were 31.2 ml/min/1.73 m^2^ (21.6–41.7 IQR) and 144 mg/g (49.0–620 IQR), respectively. Mild SHPT was highly represented [iPTH 112 pg/ml (85.4–157 IQR), PTH (1–84) 37.6 pg/ml (28.9–50.3 IQR)] in the presence of median 25(OH)D levels at the bottom threshold of normality range [31.0 ng/ml (22.7–41.8 IQR)] and normal serum levels of Ca, P, ALP, BSAP, and sclerostin. Forty (56%) patients received renin angiotensin receptor inhibitors. Vitamin D was prescribed in 46 (65%) as nutritional formulation (45%) or VDRA (3%) alone, or as combination of both nutritional and VDRA compounds (17%).

**Table 1:** Baseline characteristics and study outcomes stratified according to basal eGFR.

	**Total**	**eGFR ≥30 mL/min/1.73 m^**2**^**	**eGFR <30 mL/min/1.73 m^**2**^**	***P-*value**
Number of patients	71	38 (54	33 (46)	
Age (years)	75 (69–80)	75 (68–80)	75 (71–81)	0.53
Elderly (age > 70 years)	52 (73)	27 (71)	25 (76)	0.66
Gender (males)	54 (76)	32 (84)	22 (67)	0.09
Cardiovascular disease	50 (70)	25 (66)	25 (76)	0.36
Diabetes	30 (39)	18 (47)	12 (36)	0.35
**CKD etiology**				
Diabetes	24 (34)	14 (37)	10 (30)	0.56
Hypertension	63 (89)	33 (87)	30 (91)	0.59
Glomerulonephritis	6 (9)	5 (13)	1 (3)	0.13
Inherited diseases	2 (3)	1 (3)	1 (3)	0.92
CAKUT	35 (49)	16 (42)	19 (58)	0.20
Autoimmune diseases	5 (7)	3 (8)	2 (6)	0.76
Obstructions	11 (16)	6 (16)	5 (15)	0.94
Repeated urinary infections	6 (9)	1 (3)	5 (15)	0.06
Other	21 (30)	12 (32)	9 (27)	0.69
BMI (Kg/m^2^)	27.3 (24.5–32.2)	27.2 (24.1–30.9)	28.5 (24.9–32.3)	0.50
SBP (mmHg)	140 (120–150)	140 (130–150)	135 (120–156)	0.67
DBP (mmHg)	70 (65–80)	70 (70–80)	70 (61–80)	0.88
RAAS inhibitor	40 (56%)	24 (63%)	16 (48%)	0.22
Serum creatinine (mg/l)	1.9 (1.6–2.7)	1.6 (1.4–1.7)	2.7 (2.2–2.9)	<0.0001
eGFR (ml/min/1.73 m^2^)	31.2 (21.6–41.7)	40.9 (34.9–46.8)	21.1 (18.6–27.1)	<0.0001
mGFR (ml/min)	35.0 (25.0–45.3)	44.5 (37.0–50.0)	25.0 (19.8–30.5)	<0.0001
uACR (mg/g)	144 (49.0–620)	98.8 (42.0–303)	210 (57.5–776)	0.10
Urinary sodium (mEq/24 h)	94.0 (77.5–113)	95.0 (81.0–130)	93.0 (72.5–108)	0.23
Total serum calcium (mg/dl)	9.4 (9.2–9.8)	9.5 (9.3–9.8)	9.4 (9.0–9.7)	0.20
Serum phosphate (mg/dl)	3.5 (3.2–3.9)	3.3 (3.0–3.8)	3.6 (3.5–4.3)	0.002
iPTH (pg/ml)	112 (85.4–157)	95.8 (64.3–135)	141 (97.5–222)	0.001
PTH (1–84) (pg/ml)	37.6 (28.9–50.3)	32.0 (22.2–45.6)	42.7 (36.4–62.5)	0.002
ALP (IU/L)	75.0 (61.0–86.5)	73.5 (58.0–87.0)	79.0 (64.0–86.3)	0.41
BSAP (μg/L)	16.3 (13.3–20.3)	16.0 (12.0–21.7)	16.3 (13.8–19.3)	0.78
25(OH)D (ng/ml)	31.0 (22.7–41.8)	27.5 (20.0–41.1)	37.3 (29.2–42.3)	0.05
1,25(OH)_2_D (pg/ml)	29.9 (25.3–38.8)	31.6 (27.9–40.0)	28.8 (23.0–35.0)	0.13
iFGF-23 (pg/ml)	63.9 (47.9–88.4)	50.29 (43.2–64.7)	88.4 (60.0–120)	0.0001
Sclerostin (pg/ml)	564 (446–705)	568 (443–698)	559 (445–763)	0.89
1,25(OH)_2_D/PTH (1–84) ratio	0.74 (0.57–1.2)	0.97 (0.67–1.3)	0.56 (0.46–0.84)	<0.0001
1,25(OH)_2_D/iFGF-23 ratio	0.51 (0.31–0.76)	0.63 (0.49–0.81)	0.34 (0.22–0.58)	0.0006
Urinary phosphate (g/24 h)	0.61 (0.47–0.72)	0.65 (0.53–0.73)	0.51 (0.39–0.69)	0.04
Urinary calcium (mg/24 h)	36.2 (8–65.3)	50.2 (21.4–84.9)	24.3 (0–44.0)	0.03
**Vitamin D supplements**				0.001
Any	46 (65)	51 (47)	28 (85)	
Only nutritional	32 (45)	16 (42)	16 (49)	
Only VDRA	2 (3)	0 (-)	2 (6)	
Nutritional & VDRA	12 (17)	2 (5)	10 (30)	
WRF ≥20% at 6 months	15 (21)	6 (16)	9 (27)	0.24
iPTH increase ≥20% at 6 months	29 (41)	19(50)	10(30)	0.12
PTH (1–84) increase ≥20% at 6 months	40 (56)	23 (61)	17 (52)	0.45

*ALP, total alkaline phosphatase; BMI, body mass index; BSAP, bone specific alkaline phosphatase; CAKUT, Congenital anomalies of kidney and urinary tract; CKD, chronic kidney disease; DBP, diastolic blood pressure; eGFR, estimated glomerular filtration rate; iFGF-23, intact fibroblast growth factor – 23; iPTH, intact parathormone; mGFR, measured glomerular filtration rate; PTH (1–84), biologically active PTH (1–84); RAAS, renin angiotensin aldosterone system; SBP, systolic blood pressure; VDRA, vitamin D receptor activator; uACR, urine albumin creatinine ratio; WRF, worsening renal function*.

**Figure 1:**
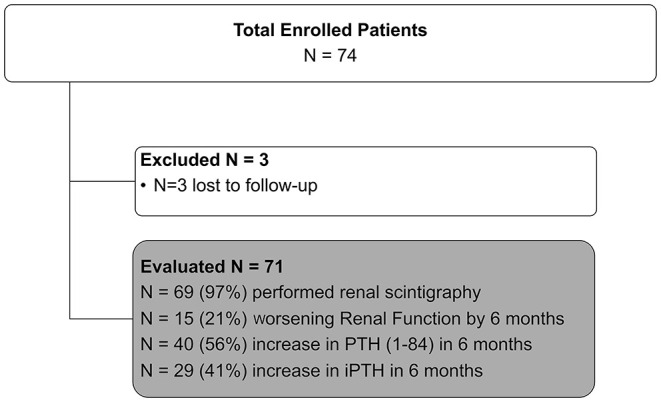
PASCaL clinical trial enrollment flow chart with endpoint frequencies.

At 6-month follow up 29 (41%) and 40 (56%) patients reached 20% increase in iPTH and PTH (1–84), respectively. Fifteen (21%) patients developed 20% WRF, respectively (Figure 1).

Basal eGFR <30 ml/min/1.73 m^2^ was associated with significantly higher levels of serum P, iPTH, PTH (1–84), iFGF-23 and lower 24 h renal excretion of both Ca and P (Table 1). The 1,25(OH)_2_D/iPTH and 1,25(OH)_2_D/PTH (1–84) ratios were significantly lower in patients with eGFR <30 ml/min/1.73_2_ (*p* < 0.001), despite similar 1,25(OH)_2_D levels between eGFR strata (Table 1). Vitamin D was more frequently prescribed within lower eGFR group (85 vs. 47%) (*p*- for trend = 0.001). Rates of WRF and PTH increase were not influenced by the basal eGFR.

Basal PTH (1–84) was lower among patients who further achieved ≥20% increase in PTH (1–84) at 6 months [34.3 pg/ml (21.7–45.9 IQR) vs. 41.5 pg/ml (32.0–58.4 IQR), *p* = 0.01] (Supplementary Table 1). Absolute PTH (1–84) increase was independently predicted by unitary lower 1,25(OH)_2_D levels (*p* = 0.0134), absence of diabetes (*p* = 0.017), older age (*p* = 0.022), and higher uACR (*p* = 0.023) (Table 2). However, 1,25(OH)_2_D taken as categorical variable (cut-off set at 29 pg/ml) was unable to predict PTH increase (data not shown). None of the basal parameters was retained for predicting 20% PTH (1–84) increase in multivariate models. The increase in iPTH levels was not predicted by 1,25(OH)_2_D as continuous or categorical variable (Supplementary Table 2). Rate of iPTH and PTH (1–84) increase was not associated with WRF (Supplementary Table 3).

**Table 2:** Multivariate regression model for predicting absolute PTH (1–84) increase at 6 months.

**Variables**	**Coefficient**	**SE**	***Z* value**	***P*-value**
Age	0.247	0.105	2.35	0.022
Diabetes	−8.66	3.52	−2.46	0.017
uACR	0.0026	0.0011	2.34	0.023
1,25(OH)_2_D	−0.317	0.124	−2.55	0.0134
Sclerostin	−0.015	0.008	−1.83	0.073

*uACR, urine albumin creatinine ratio*.

Lower baseline 1,25(OH)_2_D was the only patients' characteristics descriptively associated with WRF [25.7 pg/ml (16.2–28.9 IQR) vs. 31.4 pg/ml (28.1–39.4 IQR), respectively, *p* = 0.001] (Supplementary Table 3). At univariate regression model, unitary increase in basal 1,25(OH)_2_D levels was associated with 11% risk reduction for developing WRF [OR 0.89 (95% CI 0.86–0.93), *p* = 0.006] (Table 3). Higher 1,25(OH)_2_D/PTH (1–84) ratio was associated with 84% risk reduction for reaching the renal outcome at the limit of statistical significance [OR 0.16 (95% CI 0.06–0.41), *p* = 0.05]. Basal 1,25(OH)_2_D levels *leq* 29.0 pg/ml resulted moderately sensitive (86.7%) and poorly specific (66%) in predicting WRF in the whole cohort (*p* = 0.0002) (Table 4). Notably, sensitivity reached 100% together with mild improvement in specificity among patients with higher eGFR and lower age. On the contrary, 1,25(OH)_2_D was unreliable in predicting WRF in CKD stage 4 to 5. Basal eGFR di not predict WRF in the whole and the stratified cohorts (Table 4).

**Table 3:** Univariate regression models for predicting WRF >20% at 6 months.

**Variable**	**Coefficient**	**SE**	***Z* value**	**OR (95% CI)**	***P*-value**
eGFR (mL/min/1.73 m^2^)	−0.002	0.023	−0.08	1.00 (0.98–1.02)	0.93
uACR (mg/g)	0.0003	0.0003	1.06	1.00 (1.00–1.00)	0.29
Urinary sodium (mEq/L)	0.014	0.009	1.58	1.01 (1.01–1.02)	0.11
Total serum calcium (mg/dl)	0.126	0.738	0.18	1.13 (0.54–2.37)	0.86
Serum phosphate (mg/dl)	−0.078	0.442	−0.17	0.93 (0.59–1.44)	0.86
iPTH (pg/ml)	−0.002	0.004	−0.45	1.00 (0.99–1.00)	0.65
PTH (1–84) (pg/ml)	−0.016	0.016	−0.97	0.98 (0.97–1.00)	0.33
ALP (IU/L)	0.028	0.015	1.89	1.03 (1.01–1.04)	0.06
BSAP (g/L)	0.039	0.039	1.00	1.04 (1.00–1.08)	0.32
25(OH)D (ng/ml)	−0.031	0.024	−1.28	0.97 (0.95–0.99)	0.20
1,25(OH)_2_D (pg/ml)	−0.111	0.040	−2.74	0.89 (0.86–0.93)	0.006
iFGF-23 (pg/ml)	0.002	0.006	0.30	1.00 (1.00–1.01)	0.77
Sclerostin	−0.0010	0.001	−0.71	1.00 (1.00–1.00)	0.48
1,25(OH)_2_D/PTH (1–84) ratio	−1.85	0.95	−1.94	0.16 (0.06–0.41)	0.05
1,25(OH)_2_D/iFGF-23 ratio	0.254	0.536	0.47	1.29 (0.75–2.20)	0.64
Urinary phosphate (g/24 h)	−1.507	1.37	−1.10	0.22 (0.06–0.87)	0.27
Urinary calcium (mg/24 h)	−0.009	0.008	−1.13	0.99 (0.98–1.00)	0.26

*ALP, total alkaline phosphatase; BSAP, bone specific alkaline phosphatase; eGFR, estimated glomerular filtration rate; iFGF-23, intact fibroblast growth factor – 23; iPTH, intact parathormone; PTH (1–84), biologically active PTH (1–84); uACR, urine albumin creatinine ratio*.

**Table 4:** ROC analysis for predicting WRF ≥20% at 6 months.

	**Subgroup**	***N***	**AUC**	**95% CI**	**Criterion**	**Sensitivity**	**Specificity**	***P***
**1,25(OH)**_**2**_ **D**	All patients	71	0.773	0.658–0.864	≤ 29.0 pg/mL	86.7	66.0	0.0002
	eGFR ≥30 mL/min/1.73 m^2^	38	0.909	0.770–0.978	≤ 29.0 pg/mL	100	75.0	<0.0001
	eGFR <30 mL/min/1.73 m^2^	33	0.653	0.468–0.809	≤ 17.8 pg/mL	33.3	100	0.21
	≤ 70 years	19	0.883	0.654–0.983	≤ 27.8 pg/mL	100	73.3	<0.0001
	>70 years	52	0.734	0.593–0.847	≤ 29.0 pg/mL	81.8	65.9	0.01
**eGFR**	All patients	71	0.514	0.392–0.634	≤ 31.8 ml/min/1.73 m^2^	66.7	51.8	0.87
	eGFR ≥30 ml/min/1.73 m^2^	38	0.602	0.430–0.756	>50.9 ml/min/1.73 m^2^	50.0	84.4	0.48
	eGFR <30 ml/min/1.73 m^2^	33	0.690	0.505–0.839	>23.0 ml/min/1.73 m^2^	55.6	79.2	0.07
	≤ 70 years	19	0.583	0.339–0.801	≤ 31.8 ml/min/1.73 m^2^	75.0	60.0	0.65
	>70 years	52	0.513	0.371–0.654	>20.4 ml/min/1.73 m^2^	100	22.0	0.89

*AUC, area under the curve; eGFR, estimated glomerular filtration rate; ROC, receiving operating curve analysis*.

**Figure 2:**
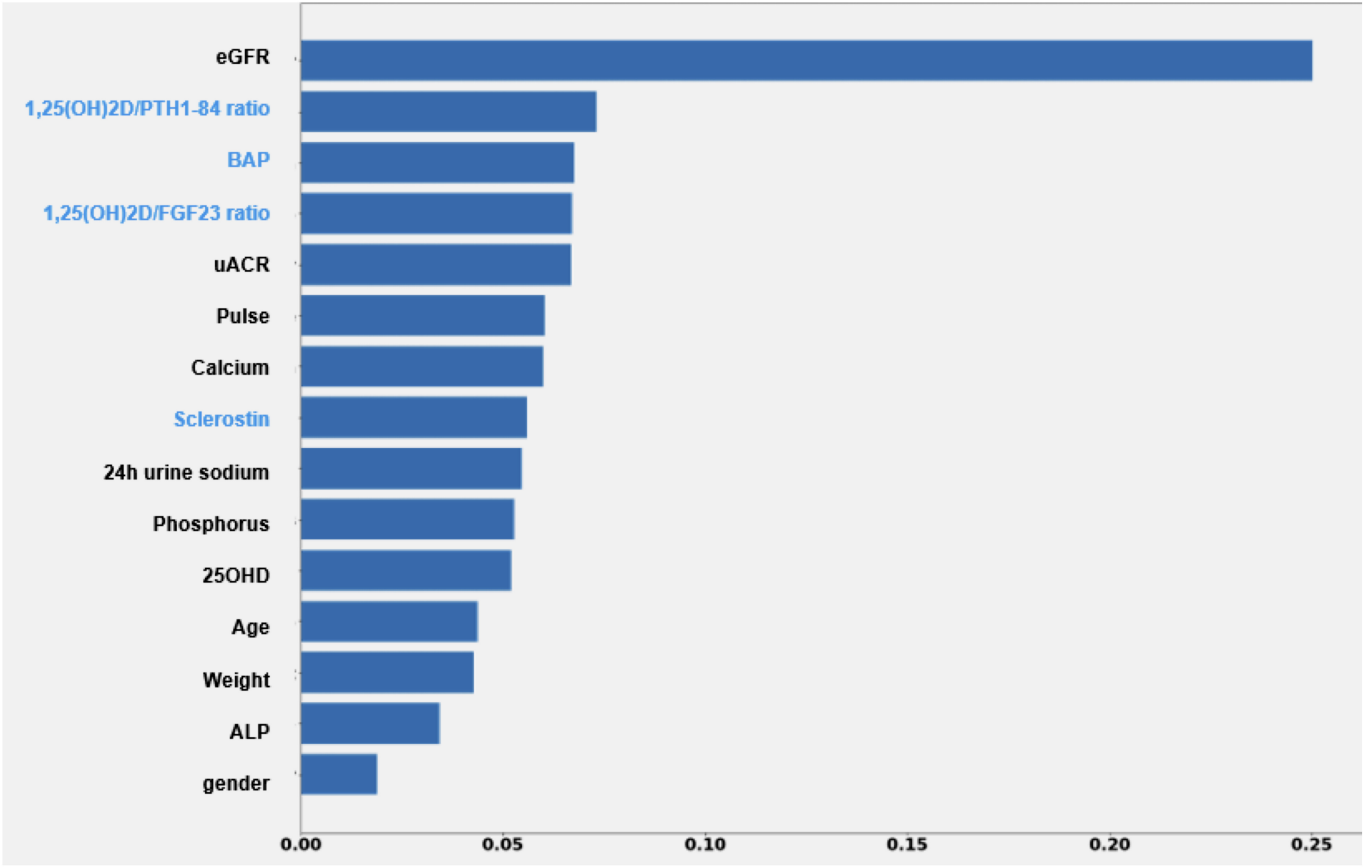
Impact of each feature of the XGBoost model on the prediction of eGFR value after 3 months. Each importance score attempts to quantify the relative importance of each feature in the prediction of the output variable. ALP, alkaline phosphatase; BAP, bone specific alkaline phosphatase; eGFR, estimated glomerular filtration rate; UACR, urine albumin creatinine ratio.

ML model, limited to standard clinical data, had a MAPE of 29.1% in predicting 3-months eGFR (Figure 2). MAPE reduced by 3.8% after inclusion of bone and mineral markers. The 1,25(OH)_2_D/PTH (1–84) ratio was retained as informative factor on renal outcome with the stronger weight, only second to eGFR independently from albuminuria. MAPE was even lower in patients younger than 70 years (MAPE = 23.2%) and with eGFR ≥ 30 ml/min/1.73 m^2^ (MAPE = 23.9%) (data not shown).

**Figure 3:**
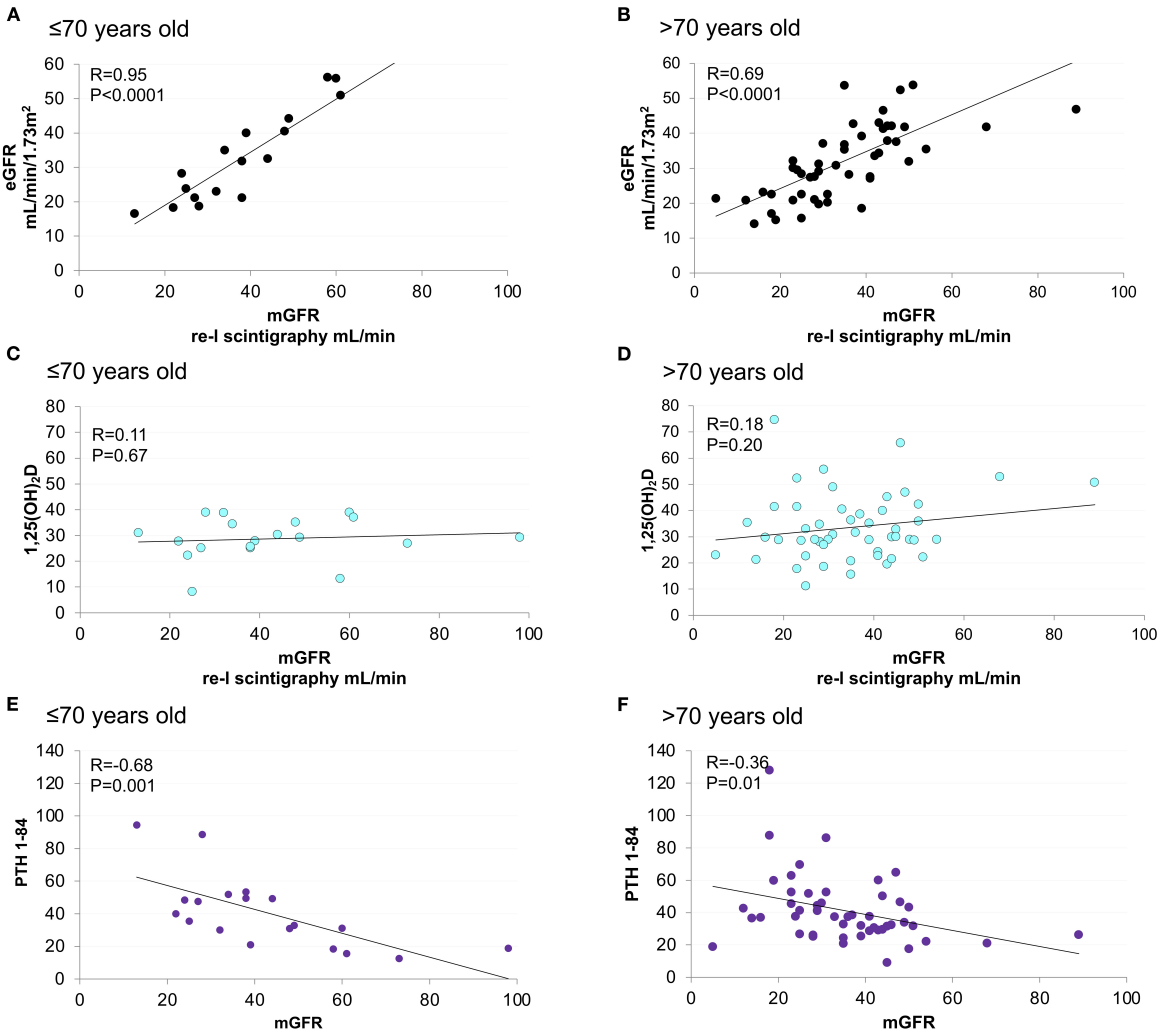
Linear correlation between mGFR and eGFR, 1,25(OH)2D and PTH (1–84), stratified according to age. Age < = 70 years old figures **(A,C,E)**. Age > 70 years old figures **(B,D,F)**.

Renal scintigraphy was performed in 69 (97%) patients. Measured GFR was linearly correlated with both eGFR and PTH (1–84) (Figure 3). The strength of correlation was attenuated among elderly patients. No correlation was detected between 1,25(OH)_2_D, mGFR, and mineral parameters at baseline (Figure 3, Supplementary Figure 1). Moderate correlation was observed between GFR and mineral parameters in patients aged ≤ 70 years.

At 6-month iFGF-23, and sclerostin significantly increased, while BSAP, 1,25(OH)_2_D/PTH (1–84) and 1,25(OH)_2_D/iFGF-23 ratios were reduced (Table 5, Supplementary Table 4). Sclerostin increase was more pronounced in patients undergoing vitamin D therapy (Supplementary Table 4).

**Table 5:** Changes in renal and mineral parameters between baseline and 6-months follow up, stratified according to basal eGFR.

**Variable**	**Whole cohort** **(*****N*** **=** **71)**				**eGFR** **≥30 mL/min/1.73 m**^**2**^ **(*****N*** **=** **38)**				**eGFR** ** <30 mL/min/1.73 m**^**2**^ **(*****N*** **=33)**				***P*-value** **for trend** **between eGFR strata**
	***N***	**Baseline**	**6 months**	***P*-value**	***N***	**Baseline**	**6 months**	***P*-value**	***N***	**Baseline**	**6 months**	***P*-value**	
		**Mean (95% CI)**	**Mean (95% CI)**			**Mean (95% CI)**	**Mean (95% CI)**			**Mean (95% CI)**	**Mean (95% CI)**		
eGFR (ml/min/1.73 m^2^)	71	33.0 (29.9–36.1)	31.6 (28.5–34.7)	0.05	38	42.5 (39.2–45.9)	40.8 (37.3–44.3)	0.08	33	22.0 (20.4–23.6)	21.0 (18.9–23.1)	0.31	0.75
Serum creatinine (mg/dl)	71	2.0 (1.8–2.1)	2.1 (1.9–2.3)	0.007	38	1.6 (1.5–1.7)	1.7 (1.6–1.8)	0.02	33	2.6 (2.4–2.8)	2.7 (2.5–2.9)	0.17	0.39
uACR (mg/g)	62	150 (99–227)	127 (84–194)	0.12	33	108 (61–189)	88 (49–158)	0.20	29	217 (117–405)	195 (107–353)	0.38	0.74
Urinary sodium (mEq/24 h)	60	95.5 (88.2–103)	96.0 (90.0–104)	0.86	35	96.7 (86.4–108)	97.3 (87.3–108)	0.90	25	93.8 (83.5–105)	94.3 (84.3–106)	0.90	0.50
Total serum Calcium (mg/dl)	71	9.4 (9.4–9.5)	9.2 (9–0-9.6)	0.17	38	9.5 (9.4–9.6)	9.5 (9.3–9.6)	0.37	33	9.4 (9.2–9.5)	9.0 (8.4–9.6)	0.22	0.71
Serum phosphate (mg/dl)	71	3.6 (3.4–3.7)	3.6 (3.5–3.8)	0.29	38	3.4 (3.2–3.5)	3.4 (3.2–3.6)	0.50	33	3.8 (3.6–4.1)	3.9 (3.7–4.1)	0.42	0.78
iPTH (pg/ml)	68	113 (100–129)	122 (106–140)	0.16	37	93.9 (80.4–110)	99.0 (82.5–119)	0.53	31	142 (118–171)	157 (130–189)	0.06	0.91
PTH (1–84) (pg/ml)	71	37.6 (33.5–42.2)	47.4 (42.3–53.0)	<0.0001	38	31.3 (27.0–36.2)	40.2 (34.9–46.3)	0.0008	33	46.5 (39.6–54.6)	57.2 (48.6–67.5)	0.003	0.78
ALP (IU/l)	71	73.1 (68.3–78.1)	74.0 (69.1–79.3)	0.46	38	70.1 (63.3–77.6)	71.8 (64.6–79.7)	0.26	33	76.6 (70.2–83.7)	76.6 (70.0–83.9)	0.99	0.52
BSAP (μg/l)	70	16.4 (15.0–17.9)	13.9 (12.7–15.2)	<0.0001	37	16.1 (13.8–18.8)	14.2 (12.2–16.4)	0.0002	33	16.6 (15.1–18.3)	13.6 (12.5–14.9)	<0.0001	0.32
25(OH)D (ng/ml)	70	30.5 (27.5–33.9)	28.7 (25.8-32.0)	0.20	37	27.6 (23.4–32.6)	26.4 (22.6–30.8)	0.51	33	34.1 (30.3–38.4)	31.6 (27.2–36.8)	0.24	0.69
1,25(OH)_2_D (pg/ml)	70	30.4 (27.7–33.2)	28.6 (25.8–31.7)	0.07	37	32.2 (28.8–36.1)	29.7 (26.4–33.5)	0.07	33	28.4 (24.5–32.9)	27.4 (23.0–32.7)	0.47	0.22
iFGF-23 (pg/ml)	68	64.8 (56.8–73.9)	79.4 (69.0–91.2)	0.0001	36	52.8 (46.9–59.5)	60.7 (53.8–68.5)	0.04	32	81.5 (65.0–102)	107 (85.5–134)	0.0008	0.06
Sclerostin (pg/ml)	70	568 (524–616)	737 (670–812)	<0.0001	37	567 (515–624)	706 (632–788)	<0.0001	33	569 (494–656)	774 (654–916)	<0.0001	0.07
1,25(OH)_2_D/PTH1-84 ratio	70	0.81 (0.71–0.92)	0.60 (0.54–0.68)	<0.0001	37	1.04 (0.89–1.2)	0.75 (0.65–0.86)	0.0002	33	0.61 (0.52–0.72)	0.48 (0.40–0.58)	0.005	0.09
1,25(OH)_2_D/iFGF-23 ratio	67	0.47 (0.39–0.56)	0.36 (0.30–0.44)	0.0001	35	0.61 (0.53–0.71)	0.49 (0.42–0.58)	0.009	32	0.35 (0.25–0.48)	0.26 (0.18–0.36)	0.005	0.53
Urinary phosphate (g/24 h)	56	0.59 (0.52–0.66)	0.62 (0.52–0.75)	0.45	32	0.62 (0.54–0.72)	0.65 (0.57–0.75)	0.50	24	0.54 (0.44–0.66)	0.59 (0.39–0.88)	0.62	0.19
Urinary calcium (mg/24 h)	57	47.0 (33.2–60.9)	50.2 (35.1–65.3)	0.46	33	58.2 (37.9–78.6)	67.1 (44.4–89.7)	0.18	24	31.6 (14.6–48.7)	27.0 (12.0–42.0)	0.34	0.10

*ALP, total alkaline phosphatase; BSAP, bone specific alkaline phosphatase; eGFR, estimated glomerular filtration rate; iFGF-23, intact fibroblast growth factor – 23; iPTH, intact parathormone; PTH (1–84), biologically active PTH (1–84); uACR, urine albumin creatinine ratio*.

## Discussion

CKD diagnosis and staging are currently based on eGFR and uACR (6, 40). However, eGFR and uACR are informative on glomerular rather than on tubular function/injury, which has been linked to the risk of CKD progression (41–43). Furthermore, tubular fibrosis was not associated with GFR, limiting its predictability by glomerular markers (44).

A growing body of evidence supports new biomarkers of tubular damage as independent predictors of WRF (12, 15, 16, 45, 46). Few molecules were classified as reliable markers of tubular *injury, inflammation/fibrosis* and *repair* (14, 46–48). More recently, biomarkers of tubular *reabsorption* and *reserve* (UMOD) were added into the panel, and factorial analysis was applied for aggregating single markers into clusters, based on their pathophysiologic role and cumulative association with clinical outcomes (13, 48, 49). FGF-23 and PTH were finally included into a single tubule *reserve/mineral metabolism* factor, together with UMOD, as biomarkers of tubular *response to hormones* (13, 49). *Post-hoc* analysis of the SPRINT trial observed that UMOD, FGF-23, PTH factor was associated with faster WRF independently from basal eGFR and albuminuria (13). The same factor was associated with increased risk of cardiovascular disease and heart failure in the same cohort (49). Hence, non-invasive tubular health assessment, including biomarkers of structural damage and cellular function, was suggested as important tool for better stratifying the risk of CKD progression (47).

Several characteristics may link UMOD to 1,25(OH)_2_D as protective biomarkers of tubular health. UMOD, otherwise known as Tamm-Horsfall protein, is expressed in the thick ascending limb of the loop of Henle and early distal convoluted tubule (50). UMOD is supposed to protect renal tubules from kidney stone formation (51), urinary tract infection (52), and ischemia (53). Higher UMOD levels were associated with lower risk of kidney function decline (14, 45, 50, 54) and were suggested as biomarker of tubular mass (45) and tubular pathology (55). Active vitamin D is synthesized in renal tubule, and deserves protective functions against inflammatory, fibrotic and oxidative insults (56). Low levels of 1,25(OH)_2_D trigger SHPT while VDRA administration improved albuminuria in experimental and clinical studies (57, 58). Hence, the pathophysiology of SHPT, the relevance of tubular health in CKD and the renal protection elicited by VDR activation support 1,25(OH)_2_D as both marker of tubular synthesis and favorable predictor of WRF, nonetheless responsive to adaptive hormonal feed-backs in the context of CKD-MBD. However, predictability of WRF by serum 1,25(OH)_2_D remains unexplored up to date.

In the PASCaL-1,25D study 1,25(OH)_2_D levels were not correlated with mGFR at baseline (Figure 2). However, higher 1,25(OH)_2_D was associated with 11% risk reduction in 6-months WRF at univariate analysis (*p* = 0.006) (Table 3). Renal outcome was not associated with any other variable. Furthermore, basal 1,25(OH)_2_D lower than 29.0 pg/ml was highly sensitive in predicting WRF in the whole cohort, reaching 100% sensitivity in younger patients and in those with better renal function at study entry (Table 4). Unfortunately, independency of association was impossible to be tested by multivariate regression models due to low rate of renal events.

Assessing biologically active PTH is relevant to analyze the clinical impact of mineral derangement in CKD. However, the most commonly used second-generation PTH assay, erroneously called iPTH, detects both the full-length biologically active PTH (1–84) and the large C-terminal PTH fragments, otherwise named “non-(1–84) PTH” (24). C-terminal PTH fragments accumulate in CKD (59), where they account for up to 50% of PTH levels assessed by second-generation assay (60) and participate to PTH resistance mediated by uremic toxins (61). On the contrary, third-generation assays detects biologically active PTH (1–84) (62). Compared with iPTH, biologically active PTH (1–84) was commonly 50–70% lower and more reliably associated with bone health in renal patients (63). However, PTH (1–84) is not routinely assessed in clinical practice, and its association with 1,25(OH)_2_D has been minimally investigated.

In the present study baseline PTH (1–84) was 2–4 times lower than iPTH (Table 1). Of note, the rate of 20% increase in PTH levels was descriptively higher when referred to PTH (1–84) (56%) compared with iPTH (41%) independently from eGFR strata (Table 1). Although 1,25(OH)_2_D and PTH were not correlated at baseline (Supplementary Figure 1), 1,25(OH)_2_D independently predicted PTH (1–84) (but not iPTH) increase at 6 months (Table 2, Supplementary Table 2), suggesting that lower 1,25(OH)_2_D levels may help in recognizing patients at increased risk of progression toward biologically-relevant SHPT.

Vitamin D deficiency and SHPT were associated with the risk of CKD progression in observational studies (64, 65). SHPT alters bone turnover and mineralization, and participates to vascular and renal aging, mediated by P and FGF-23 toxicity (66). Based on physiology driven approach, the lower the 1,25(OH)_2_D and the higher the biologically active PTH, the most maladaptive could be the endocrine response to CKD-MBD. The 1,25(OH)_2_D/PTH ratio was previously tested as negative predictor of CKD progression in *post-hoc* analysis from the GISSI-HF and CanPREDDICT studies. The GISSI-HF trial was a randomized control study, conducted among 6.975 patients with stable heart failure (67). The study included middle aged patients (66.8 ± 10.8 years) with mild to moderate CKD (eGFR 68.6 + 23.5 ml/min/1.73 m^2^) (29). In *post-hoc* analysis by Masson et al. (29) 335 (29.6%) experienced WRF, defined as ≥0.3 mg/dl and ≥25% increase in sCr at two consecutive study visits during 60-month follow up. Lower 1,25(OH)_2_D/PTH (1–84) ratio was independently associated with 25 and 35% risk reduction for the renal outcome and all-cause mortality, respectively. Notably, a weaker association was detected when taking 1,25(OH)_2_D and PTH (1–84) as individual predictors. In the present study older age, stronger severity of CKD, SHPT, and 1,25(OH)_2_D depletion may account for significant association between WRF and 1,25(OH)_2_D taken as single predictor. The observational CanPREDDICT study was conducted in Canada among CKD stage 3–4 patients (68) with demographic and renal characteristics comparable with the PASCaL-1,25D study. In the *post-hoc* analysis, conducted by Levin A. et al. among 1.784 patients, lower 1,25(OH)_2_D and lower 1,25(OH)2D/PTH (1–84) ratio were associated with 50% decline in eGFR over 5-years follow up independently from albuminuria (30).

In the PASCAL-1,25D study 1,25(OH)2D/PTH (1–84) ratio was significantly lower in patients with eGFR <30 ml/min/1.73 m^2^ at baseline, mainly attributable to higher PTH (1–84) levels (Table 1). Higher 1,25(OH)_2_D/PTH (1–84) ratio was associated with 84% reduction in the risk for WRF at the limit of statistical significance at univariate analysis (Table 3). Although regression models did not retain albuminuria and eGFR as predictors of renal outcome, ML analysis selected 1,25(OH)_2_D/PTH (1–84) as informative factor on 3-months eGFR prediction with the best relevance only secondary to the baseline eGFR. Albuminuria was retained in the same model, although with mildly lower weight compared with 1,25(OH)_2_D/PTH (1–84) ratio (Figure 2). The small sample size, the low rate of renal events and the shortness of follow up in the PASCAL-1,25D study may partially explain discrepant results obtained by classic statistics compared with ML approach.

Interpretation of biomarkers must be contextualized to clinical scenarios and patients' characteristics (48). Although elderly accounted for more than 70% of the entire cohort, younger patients deserved the strongest association between mineral and glomerular biomarkers (Figure 3, Supplementary Figure 1) as the more accurate predictability of WRF by 1,25(OH)_2_D (Table 4). This reminds the urgent need of further studies to investigate peculiarities of CKD-MBD in the elderly (69).

Several limitations hampered the PASCaL-1,25D study, including small sample size, short follow-up, low achievement of renal outcome, uncontrolled vitamin D administration, and unavailable assessment of other tubule biomarkers and oxidated PTH (25). Furthermore, comparisons between present data and *post-hoc* analysis from the SPRINT trial must be taken cautiously, due to exclusion of patients with diabetes and proteinuria > 1 g/24 h from that study (70). Present data remain underpowered to orient decisions on vitamin D therapy in CKD.

In conclusion, PASCaL-1,25D study suggests 1,25(OH)_2_D as a promising biomarker of tubular health, and sensible predictor of WRF on the short run among non-elderly patients affected by stage-3 CKD. The 1,25(OH)_2_D/PTH (1–84) ratio might represent a composite biomarker of tubular reserve/endocrine response to the transition from adaptive to maladaptive equilibrium in CKD-MBD. The aforesaid conclusions must be taken as hypothetical, in expectance of validation by further research.

## Data Availability Statement

The raw data supporting the conclusions of this article will be made available by the authors, without undue reservation.

## Ethics Statement

The studies involving human participants were reviewed and approved by Comitato Etico Interaziendale Milano Area A. The patients/participants provided their written informed consent to participate in this study.

## Author Contributions

AG and MGC: concept, methodology, supervision, and editing. EF and RC: patient enrolment and data collection. MAP, CM, and PC: data analysis. FB and CZ: data analysis, writing original draft, and editing. FAB: writing original draft and editing. MC: data curation and project administration. All authors contributed to the article and approved the submitted version.

## Funding

This study was funded and supported by DiaSorin SpA manufacturer of bone and mineral biomarker assays.

## Conflict of Interest

FB, MC, and FAB are employees of DiaSorin the manufacturer of the LIAISON^®^ bone and mineral tests. CZ is an independent consultant to DiaSorin. MGC has received consulting fees from DiaSorin in the past for advisory board membership. AG and MGC have received speaking honoraria from Abbvie, Amgen, Shire, and Vifor Fresenius. CM and MAP are employed by Reply Data. The remaining authors declare that the research was conducted in the absence of any commercial or financial relationships that could be construed as a potential conflict of interest.

## Publisher's Note

All claims expressed in this article are solely those of the authors and do not necessarily represent those of their affiliated organizations, or those of the publisher, the editors and the reviewers. Any product that may be evaluated in this article, or claim that may be made by its manufacturer, is not guaranteed or endorsed by the publisher.
