# Supplementary material for: 1,25-dihydroxyvitamin D as Predictor of Renal Worsening Function in Chronic Kidney Disease. Results From the PASCaL-1,25D Study
Source: Front Med (Lausanne). 2022 Mar 2;9:840801. doi: 10.3389/fmed.2022.840801 (PMC8924653; doi:10.3389/fmed.2022.840801)
Supplement: Supplementary file 1 [file Data_Sheet_1.pdf]

S. Figure 1 Correlation matrix between mineral and renal biomarkers, according to age

1.0 – -1.0

>70 years old

	age	BMI	systolic BP	diastolic BP	mGFR	eGFR	Serum creatinine	ALP	calcium	phosphorus	24h urine phosphorus	24h urine calcium	24h urine sodium	uACR	intact PTH	PTH1-84	25OHD	$1,25(OH)_2D$	FGF23	Sclerostin	BAP	$1,25(OH)_2D$/PTH1-84 ratio	$1,25(OH)_2D$/FGF23 ratio
**age**		-0.12	-0.11	-0.31	-0.08	-0.07	-0.10	-0.07	-0.04	0.21	-0.13	-0.23	-0.27	-0.25	0.07	0.12	0.01	0.05	0.30	0.02	-0.10	0.05	-0.16
**BMI**	0.49		-0.10	0.22	-0.02	-0.18	0.18	-0.01	-0.08	0.05	0.28	0.18	-0.06	0.24	-0.02	-0.13	0.20	0.00	0.01	-0.10	0.10	0.18	0.01
**systolic BP**	0.27	0.57		0.44	0.15	0.21	-0.19	0.17	0.07	0.01	0.23	0.07	0.07	0.20	-0.07	-0.02	-0.30	0.13	-0.10	0.05	-0.05	0.10	0.08
**diastolic BP**	0.07	-0.05	0.18		0.09	-0.08	0.14	0.07	0.17	0.05	0.08	0.39	-0.05	0.14	-0.04	0.02	0.05	0.19	0.02	0.04	0.00	0.16	0.10
**mGFR**	0.07	-0.08	-0.30	0.35		0.69	-0.60	-0.10	0.12	-0.43	0.34	0.14	0.17	-0.04	-0.33	-0.36	-0.13	0.19	-0.31	-0.03	0.04	0.48	0.11
**eGFR**	0.17	0.01	-0.25	0.25	0.95		-0.88	-0.08	0.12	-0.44	0.32	-0.01	0.19	-0.02	-0.42	-0.44	-0.32	0.06	-0.45	-0.06	0.08	0.42	0.15
**Serum creatinine**	-0.41	-0.15	0.25	-0.13	-0.76	-0.89		0.15	-0.10	0.28	-0.28	-0.07	-0.14	0.06	0.38	0.35	0.21	-0.21	0.50	0.12	-0.05	-0.40	-0.28
**ALP**	-0.09	-0.25	0.02	0.16	-0.30	-0.30	0.33		-0.05	0.19	0.07	-0.22	0.12	0.04	0.27	0.24	-0.17	-0.22	0.19	0.16	0.75	-0.30	-0.21
**calcium**	0.12	0.32	-0.07	0.42	0.55	0.46	-0.39	-0.34		0.06	-0.19	0.27	0.13	-0.23	-0.25	-0.05	0.22	-0.15	-0.16	-0.13	-0.11	-0.11	0.09
**phosphorus**	0.00	0.31	0.28	-0.33	-0.64	-0.60	0.46	0.12	-0.18		-0.10	-0.14	-0.22	-0.14	0.24	0.31	0.16	-0.10	0.36	0.01	0.08	-0.26	-0.23
**24h urine phosphorus**	-0.37	0.18	0.13	0.19	0.20	0.10	0.18	-0.12	0.34	-0.46		0.10	0.03	0.11	0.01	-0.07	-0.15	0.13	-0.10	-0.23	0.31	0.16	-0.01
**24h urine calcium**	0.07	-0.10	-0.26	0.32	0.70	0.75	-0.61	-0.04	0.28	-0.60	0.28		-0.01	0.17	-0.15	-0.05	0.37	0.31	-0.18	-0.30	-0.09	0.14	0.18
**24h urine sodium**	0.51	0.15	-0.07	0.20	0.38	0.45	-0.66	-0.49	0.33	-0.32	-0.21	0.47		-0.02	-0.02	-0.08	0.08	-0.10	-0.10	-0.01	-0.07	-0.03	-0.08
**uACR**	-0.33	-0.14	0.38	0.43	-0.07	-0.11	0.29	0.25	-0.11	0.26	-0.10	-0.03	-0.31		0.10	0.02	-0.15	0.33	-0.05	-0.15	0.02	0.15	0.17
**intact PTH**	0.04	0.22	0.31	-0.43	-0.73	-0.76	0.72	0.39	-0.49	0.59	-0.05	-0.50	-0.51	-0.08		0.84	-0.15	0.33	0.22	-0.19	0.27	-0.30	-0.03
**PTH1-84**	0.06	0.20	0.29	-0.40	-0.68	-0.72	0.72	0.41	-0.43	0.55	-0.03	-0.45	-0.51	-0.10	0.99		-0.10	0.40	0.10	-0.20	0.20	-0.54	0.05
**25OHD**	-0.23	-0.12	-0.42	-0.23	-0.18	-0.19	0.12	0.04	0.00	0.27	-0.22	-0.52	-0.38	-0.11	-0.03	-0.08		-0.03	0.26	-0.05	-0.20	0.03	-0.08
**$1,25(OH)_2D$**	0.26	-0.08	-0.63	-0.20	0.11	0.03	-0.11	0.16	0.13	-0.09	-0.14	0.09	-0.02	-0.49	0.14	0.18	0.38		-0.23	-0.31	-0.13	0.37	0.56
**FGF23**	-0.24	0.15	0.59	0.17	-0.49	-0.51	0.64	0.41	-0.16	0.53	0.26	-0.45	-0.62	0.67	0.41	0.40	-0.01	-0.43		0.32	-0.05	-0.04	-0.63
**Sclerostin**	0.21	-0.15	-0.07	-0.23	-0.02	0.04	0.03	-0.04	-0.20	-0.25	0.09	-0.15	-0.18	-0.25	0.01	0.02	0.36	0.21	0.00		-0.01	-0.04	-0.14
**BAP**	-0.04	-0.30	-0.27	-0.28	-0.41	-0.32	0.15	0.67	-0.43	0.11	-0.22	0.10	-0.15	-0.09	0.34	0.33	-0.03	0.36	0.06	-0.05		-0.23	-0.14
**$1,25(OH)_2D$/PTH1-84 ratio**	0.14	0.06	-0.46	0.19	0.71	0.71	-0.68	-0.44	0.58	-0.41	0.12	0.58	0.44	-0.10	-0.73	-0.70	0.22	0.40	-0.47	0.08	-0.28		0.22
**$1,25(OH)_2D$/FGF23 ratio**	0.30	-0.10	-0.48	0.13	0.48	0.42	-0.51	-0.02	0.39	-0.46	-0.18	0.49	0.55	-0.43	-0.35	-0.30	-0.06	0.53	-0.73	-0.13	0.08	0.44	

≤70 years old

Supplementary Figure 1 legend:

- ALP: total alkaline phosphatase

- BMI: body mass index

- BSAP: bone specific alkaline phosphatase

- CAKUT: Congenital anomalies of kidney and urinary tract

- CKD: chronic kidney disease

- DBP: diastolic blood pressure

- eGFR: estimated glomerular filtration rate

- iFGF-23: intact fibroblast growth factor - 23

- iPTH: intact parathormone

- mGFR: measured glomerular filtration rate

- PTH (1-84): biologically active PTH (1-84)

- RAAS: renin angiotensin aldosterone system

- SBP: systolic blood pressure

- VDRA: vitamin D receptor activator

- uACR: urine albumin creatinine ratio

- WRF: worsening renal function

S. Table 1. Baseline characteristics stratified according to PTH (1-84) increase at 6 months

	**Total**	**PTH (1-84) increase < 20% at 6 months**	**PTH (1-84) increase ≥ 20% at 6 months**	**P-value**
Number of patients	71	31 (44)	40 (56)	
Age (years)	75 (69-80)	75 (71-81)	75 (69-80)	0.68
Elderly (age > 70 years)	52 (73)	24 (77)	28 (70)	0.49
Gender (males)	54 (76%)	23 (74%)	31 (78%)	0.75
Cardiovascular events	50 (70)	23 (74)	27 (68)	0.54
Diabetes	30 (39)	12 (39)	18 (45)	0.60
CKD etiology				
Diabetes	24 (34)	8 (26)	16 (40)	0.21
Hypertension	63 (89)	28 (90)	35 (88)	0.71
Glomerulonephritis	6 (9)	1 (3)	5 (13)	0.17
Inherited diseases	2 (3)	1 (3)	1 (3)	0.86
CAKUT	35 (49)	14 (45)	21 (53)	0.54
Autoimmune diseases	5 (7)	2 (6)	3 (8)	0.87
Obstructions	11 (16)	3 (10)	6 (16)	0.46
Repeated urinary infections	6 (9)	2 (6)	4 (10)	0.60
Other	21 (30)	9 (29)	12 (30)	0.93
BMI (Kg/m^2)	27.3 (24.5-32.2)	27.5 (24.0-32.3)	27.2 (24.8-31.7)	0.88
SBP (mmHg)	140 (120-150)	140 (120-150)	140 (123-150)	0.93
BDP (mmHg)	70 (65-80)	70 (70-80)	70 (60-80)	0.76
RAAS inhibitor	40 (56%)	17 (55%)	23 (58%)	0.82
Serum creatinine (mg/dL)	1.9 (1.6-2.7)	2.0 (1.6-2.8)	1.8 (1.6-2.6)	0.42
eGFR (mL/min/1.73m^2)	31.2 (21.6-41.7)	29.0 (20.9-41.0)	31.5 (23.0-42.0)	0.41
mGFR (mL/min)	35.0 (25.0-45.3)	37.0 (25.0-44.8)	35.0 (27.0-46.0)	0.68
eGFR ≥ 30 mL/min/1.73m^2	38 (53)	15 (48)	23 (58)	0.45
uACR (mg/g)	144 (49.0-620)	117 (52.8-348)	145 (47.5-705)	0.48
Urinary sodium (mEq/24h)	94.0 (77.5-113)	90.0 (77.8-111)	94.5 (77.5-132)	0.55
Total serum calcium (mg/dL)	9.4 (9.2-9.8)	9.5 (9.2-9.8)	9.4 (9.2-9.8)	0.83
Serum Phosphate (mg/dL)	3.5 (3.2-3.9)	3.5 (3.3-3.9)	3.5 (3.2-4.0)	0.85
iPTH (pg/mL)	112 (85.4-157)	123 (90.1-200)	104 (65.0-147)	0.13
PTH (1-84) (pg/mL)	37.6 (28.9-50.3)	41.5 (32.0-58.4)	34.3 (21.7-45.9)	0.01
ALP (IU/L)	75.0 (61.0-86.5)	76.0 (61.0-85.0)	74.0 (61.5-87.5)	0.76
BSAP (□g/L)	16.3 (13.3-20.3)	16.2 (12.2-19.6)	16.4 (13.7-21.1)	0.81
25OHD (ng/mL)	31.0 (22.7-41.8)	31.0 (26.2-42.3)	31.3 (21.4-41.8)	0.68
1,25(OH)$_2$D (pg/mL)	29.9 (25.3-38.8)	30.3 (28.3-37.8)	29.1 (24.7-38.9)	0.49
iFGF-23 (pg/mL)	63.9 (47.9-88.4)	64.7 (49.3-86.2)	62.2 (45.0-88.4)	0.67
Sclerostin (pg/mL)	564 (446-705)	565 (454-734)	552 (447-701)	0.73
1,25(OH)$_2$D / PTH (1-84) ratio	0.74 (0.57-1.2)	0.66 (0.56-0.95)	0.82 (0.60-1.3)	0.07
1,25(OH)$_2$D / iFGF-23 ratio	0.51 (0.31-0.76)	0.50 (0.34-0.69)	0.51 (0.29-0.76)	0.98
Urinary phosphate (g/24h)	0.61 (0.47-0.72)	0.64 (0.42-0.71)	0.60 (0.48-0.72)	0.77
Urinary calcium (mg/24 h)	36.2 (8-65.3)	33.3 (0-65.5)	37.2 (19.5-65.0)	0.89
Vitamin D supplements				0.23
Any	46 (65)	21 (68)	25 (63)	
Only nutritional	32 (45)	12 (39)	20 (50)	
Only VDRA	2 (3)	2 (6)	0	
Nutritional & VDRA	12 (17)	7 (23)	5 (13)	
WRF ≥ 20% at 6 months	15 (21)	5 (16)	10 (25)	0.37

S. Table 1 legend

- ALP: total alkaline phosphatase

- BMI: body mass index

- BSAP: bone specific alkaline phosphatase

- CAKUT: Congenital anomalies of kidney and urinary tract

- CKD: chronic kidney disease

- DBP: diastolic blood pressure

- eGFR: estimated glomerular filtration rate

- iFGF-23: intact fibroblast growth factor - 23

- iPTH: intact parathormone

- mGFR: measured glomerular filtration rate

- PTH (1-84): biologically active PTH (1-84)

- RAAS: renin angiotensin aldosterone system

- SBP: systolic blood pressure

- VDRA: vitamin D receptor activator

- uACR: urine albumin creatinine ratio

- WRF: worsening renal function

S. Table 2-A. Multivariate regression model for predicting 20% iPTH increase at 6 months.

Variables	coefficient	SE	Z value	P-value
Serum calcium	2.28	0.87	2.62	0.009
eGFR	0.06	0.03	2.13	0.03
uACR	0.002	0.0007	2.11	0.03

S

S. Table 2-B. Multivariate regression model for predicting absolute iPTH increase at 6 months, including 1,25(OH)$_2$D as continuous variable.

Variables	coefficient	SE	Z value	P-value
Diabetes	-21.9	9.6	-2.29	0.026
Serum calcium	20.5	11.7	1.75	0.09
uACR	0.006	0.004	1.54	0.13

S. Table 2-C. Multivariate regression model for predicting absolute iPTH increase at 6 months, including 1,25(OH)$_2$D as categorical variable.

Variables	coefficient	SE	Z value	P-value
Serum Calcium	25.4	14.1	1.80	0.08
uACR	0.01	0.004	2.28	0.03
25(OH)D	0.69	0.43	1.61	0.11

S. Table 2 legend

- eGFR: estimated glomerular filtration rate

- uACR: urine albumin creatinine ratio

S. Table 3: Baseline characteristics stratified according to 20 WRF at 6 months

	**All**	**<20% WRF**	**≥20% WRF**	**P-value**
Number of patients	71	56 (79)	15 (21)	
Age (years)	75 (69-80)	76 (69-81	74 (69-78)	0.31
Elderly (age > 70 years)	52 (73)	41 (73)	11 (73)	0.99
Gender (male)	54 (76)	42 (75)	12 (80)	0.69
Cardiovascular events	50 (70)	38 (68)	12 (80)	0.36
Diabetes	30 (39)	23 (41)	7 (47)	0.70
CKD etiology				
Diabetes	24 (34)	17 (30)	7 (47)	0.24
Hypertension	63 (89)	49 (88)	14 (93)	0.53
Glomerulonephritis	6 (9)	4 (7)	2 (13)	0.45
Inherited diseases	2 (3)	2 (4)	0	0.46
CAKUT	35 (49)	28 (50)	7 (47)	0.82
Autoimmune diseases	5 (7)	5 (9)	0	0.95
Obstructions	11 (16)	8 (14)	3 (20)	0.59
Repeated urinary infections	6 (9)	5 (9)	1 (10)	0.78
Other	21 (30)	18 (32)	3 (20)	0.36
BMI (Kg/m^2)	27.3 (24.5-32.2)	27.1 (23.9-32.3)	28.4 (25.4-31.9)	0.59
SBP (mmHg)	140 (120-150)	138 (123-150)	145 (120-158)	0.41
DBP (mmHg)	70 (65-80)	70 (65-80)	70 (60-80)	0.90
RAAS inhibitor	40 (56%)	32 (57%)	8 (53%)	0.79
Serum creatinine (mg/dL)	1.9 (1.6-2.7)	1.8 (1.6-2.7)	2.1 (1.4-2.9)	0.77
eGFR (mL/min/1.73m^2)	31.2 (21.6-41.7)	32.0 (21.2-41.5)	29.0 (22.8-40.5)	0.87
eGFR ≥ 30 mL/min/1.73m^2	38 (54)	32 (57%)	6 (40)	0.24
mGFR (mL/min)	35.0 (25.0-45.3)	37.5 (28.0-46.0)	29.0 (24.3-40.3)	0.21
uACR (mg/g)	144 (49.0-620)	144 (50.6-535)	192 (46.2-851)	0.44
Urinary sodium (mEq/24h)	94.0 (77.5-113)	93.5 (74.5-112)	95.0 (82.5-144)	0.25
Total serum calcium (mg/dL)	9.4 (9.2-9.8)	9.4 (9.2-9.8)	9.7 (9.3-9.8)	0.41
Serum phosphate (mg/dL)	3.5 (3.2-3.9)	3.6 (3.3-3.9)	3.5 (3.2-4.1)	0.70
iPTH (pg/mL)	112 (85.4-157)	108 (83.0-166)	123 (95.9-138)	0.90
PTH (1-84) (pg/mL)	37.6 (28.9-50.3)	37.6 (27.9-52.4)	37.8 (29.9-45.7)	0.77
ALP (IU/L)	75.0 (61.0-86.5)	74.5 (60.5-83.5)	85.0 (65.5-99.0)	0.09
BSAP (μg/L)	16.3 (13.3-20.3)	16.1 (12.9-19.5)	16.3 (14.0-21.3)	0.52
25(OH)D (ng/mL)	31.0 (22.7-41.8)	30.7 (24.9-43.7)	31.2 (21.5-40.1)	0.32
1,25(OH)$_2$D (pg/mL)	29.9 (25.3-38.8)	31.4 (28.1-39.4)	25.7 (16.2-28.9)	0.001
iFGF-23 (pg/mL)	63.9 (47.9-88.4)	64.6 (49.2-85.3)	60.9 (42.9-109)	0.65
Sclerostin (pg/mL)	564 (446-705)	562 (439-685)	650 (461-847)	0.50
1,25(OH)$_2$D / PTH (1-84) ratio	0.74 (0.57-1.2)	0.82 (0.58-1.3)	0.69 (0.53-0.81)	0.08
1,25(OH)$_2$D / iFGF-23 ratio	0.51 (0.31-0.76)	0.55 (0.35-0.79)	0.36 (0.27-0.61)	0.14
Urinary phosphate (g/24h)	0.61 (0.47-0.72)	0.63 (0.48-0.72)	0.50 (0.43-0.71)	0.35
Urinary calcium (mg/24 h)	36.2 (8-65.3)	36.1 (16.0-70.2)	33.4 (0-60.8)	0.49
Vitamin D supplements				0.91
Any	46 (65)	36 (64)	10 (67)	
Only nutritional	32 (45)	25 (45)	7 (47)	
Only VDRA	2 (3)	2 (4)	-	
Nutritional & VDRA	12 (17)	9 (16)	3 (20)	
iPTH increase ≥ 20% at 6 months	29 (41)	22 (39)	7 (47)	0.54
PTH (1-84) increase ≥ 20% at 6 months	40 (56)	30 (54)	10 (67)	0.37

S. Table 3 legend

alkaline phosphatase

- BMI: body mass index

- BSAP: bone specific alkaline phosphatase

- CAKUT: Congenital anomalies of kidney and urinary tract

- CKD: chronic kidney disease

- DBP: diastolic blood pressure

- eGFR: estimated glomerular filtration rate

- iFGF-23: intact fibroblast growth factor - 23

- iPTH: intact parathormone

- mGFR: measured glomerular filtration rate

- PTH (1-84): biologically active PTH (1-84)

- RAAS: renin angiotensin aldosterone system

- SBP: systolic blood pressure

- VDRA: vitamin D receptor activator

- uACR: urine albumin creatinine ratio

- WRF: worsening renal function

S. Table 4: Supplementary Table 4. Changes in renal and mineral parameters between baseline and 6-months follow up, stratified according to any Vitamin D administration

Variable	Whole cohort (N=71)				Vitamin D (N=46)				No Vitamin D (N=25)				P-value for trend between Vitamin D strata
	N	Baseline Mean (95% CI)	6 months Mean (95% CI)	P-value	N	Baseline Mean (95% CI)	6 months Mean (95% CI)	P-value	N	Baseline Mean (95% CI)	6 months Mean (95% CI)	P-value	
eGFR (mL/min/1.73m^2)	71	33.0 (29.9-36.1)	31.6 (28.5-34.7)	0.05	46	29.4 (25.9-32.9)	28.7 (25.1-32.3)	0.52	25	39.6 (34.2-45.0)	36.9 (31.3-42.5)	0.02	0.27
Serum creatinine (mg/dL)	71	2.0 (1.8-2.1)	2.1 (1.9-2.3)	0.007	46	2.1 (1.9-2.3)	2.2 (2.0-2.5)	0.06	25	1.8 (1.6-2.0)	1.9 (1.7-2.1)	0.03	0.97
uACR (mg/g)	62	150 (99-227)	127 (84-194)	0.12	40	141 (84.4-237)	134 (79.5-226)	0.69	22	166 (78.0-353)	116 (54.2-249)	0.02	0.24
Urinary sodium (mEq/24h)	60	95.5 (88.2-103)	96.0 (90.0-104)	0.86	37	90.0 (81.8-99.1)	90.7 (83.1-99.1)	0.86	23	105 (91.5-120)	105 (91.3-121)	0.96	0.81
Total serum calcium (mg/dL)	71	9.4 (9.4-9.5)	9.2 (9-0-9.6)	0.17	46	9.4 (9.3-9.6)	9.1 (8.7-9.6)	0.21	25	9.5 (9.4-9.6)	9.5 (9.3-9.6)	0.34	0.56
Phosphorus (mg/dL)	71	3.6 (3.4-3.7)	3.6 (3.5-3.8)	0.29	46	3.7 (3.6-3.9)	3.8 (3.6-4.0)	0.57	25	3.3 (3.0-3.6)	3.4 (3.1-3.6)	0.34	0.79
iPTH (pg/mL)	68	113 (100-129)	122 (106-140)	0.16	45	124 (106-145)	136 (118-158)	0.05	23	95.5 (76.9-119)	98.0 (73.3-131)	0.83	0.95
PTH (1-84) (pg/mL])	71	37.6 (33.5-42.2)	47.4 (42.3-53.0)	<0.0001	46	39.7 (34.2-46.1)	50.3 (43.7-58.0)	0.0006	25	34.0 (28.2-41.0)	42.4 (35.1-51.2)	0.002	0.80
ALP (IU/L)	71	73.1 (68.3-78.1)	74.0 (69.1-79.3)	0.46	46	74.3 (68.3-81.0)	75.6 (69.4-82.4)	0.49	25	70.7 (62.9-79.6)	71.1 (62.9-80.2)	0.81	0.76
BSAP (μg/L)	70	16.4 (15.0-17.9)	13.9 (12.7-15.2)	<0.0001	46	16.6 (14.7-18.7)	13.8 (12.4-15.3)	<0.0001	24	16.0 (13.8-18.4)	14.2 (12.0-16.8)	0.01	0.11
25(OH)D (ng/mL)	70	30.5 (27.5-33.9)	28.7 (25.8-32.0)	0.20	46	34.7 (31.3-38.5)	32.7 (29.2-36.7)	0.23	24	23.8 (19.4-29.3)	22.3 (18.3-27.3)	0.54	0.80
1,25(OH)$_2$D (pg/mL)	70	30.4 (27.7 -33.2)	28.6 (25.8-31.7)	0.07	46	31.6 (28.5 -35.1)	29.6 (26.2-33.5)	0.11	24	28.1 (23.5-33.4)	26.8 (22.1-32.4)	0.40	0.80
iFGF-23 (pg/mL)	68	64.8 (56.8-73.9)	79.4 (69.0-91.2)	0.0001	44	72.4 (61.1-85.9)	88.3 (74.3-105)	0.002	24	52.8 (43.6-64.1)	65.2 (51.7-82.3)	0.03	0.96
Sclerostin (pg/mL)	70	568 (524-616)	737 (670-812)	<0.0001	46	582 (524-648)	786 (702-881)	<0.0001	24	542 (475-618)	652 (545-780)	0.004	0.03
1,25(OH)$_2$D / PTH (1-84) ratio	70	0.81 (0.71-0.92)	0.60 (0.54-0.68)]	<0.0001	46	0.80 (0.67-0.94)	0.59 (0.50-0.70)	0.0004	24	0.84 (0.68-1.04)	0.64 (0.53-0.76)	0.0005	0.78
1,25(OH)$_2$D / iFGF-23 ratio	67	0.47 (0.39-0.56)	0.36 (0.30-0.44)	0.0001	44	0.43 (0.34-0.55)	0.34 (0.27-0.43)	0.003	23	0.53 (0.40-0.72)	0.41 (0.29-0.58)	0.01	0.41
Urinary phosphate (g/24h)	56	0.59 (0.52-0.66)	0.62 (0.52-0.75)	0.45	34	0.56 (0.48-0.66)	0.61 (0.46-0.82)	0.52	22	0.63 (0.53-0.74)	0.64 (0.55-0.75)	0.62	0.76
Urinary calcium (mg/24h)	57	47.0 (33.2-60.9)	50.2 (35.1-65.3)	0.46	35	38.6 (21.9-55.3)	36.8 (20.2-53.3)	0.63	22	60.5 (35.6-85.4)	71.6 (43.0-100)	0.24	0.26

S. Table 4 legend

ALP: total alkaline phosphatase

- BSAP: bone specific alkaline phosphatase
- eGFR: estimated glomerular filtration rate
- iFGF-23: intact fibroblast growth factor - 23
- iPTH: intact parathormone
- PTH (1-84): biologically active PTH (1-84)
- uACR: urine albumin creatinine ratio
